# Supplementary material for: Mechanochemical Synthesis of Polyanilines and Their Nanocomposites: A Critical Review
Source: Polymers (Basel). 2022 Dec 28;15(1):133. doi: 10.3390/polym15010133 (PMC9823481; doi:10.3390/polym15010133)
Supplement: Supplementary file 1 [file polymers-15-00133-s001.zip › polymers-2037028-supplementary.pdf]

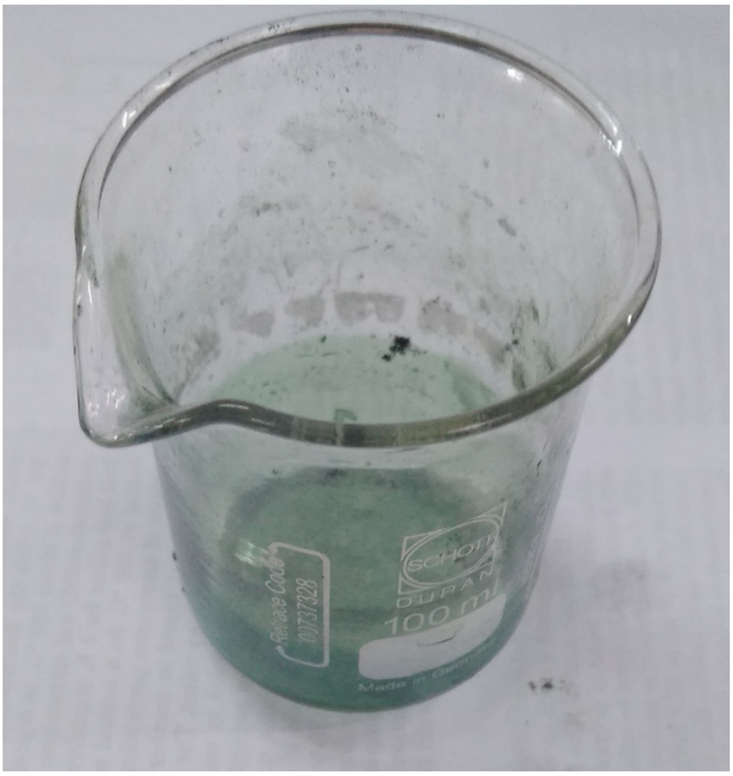

Figure S1.
Beaker where the grinded (15 min) mixture of AniHCl and APS was mixed with 50 ml water, then removed for filtration. The PANI film is formed on glass because the reaction was not complete during grinding and is finished in the solution.

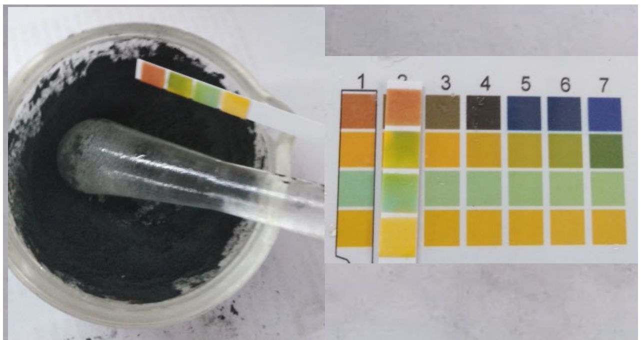

Figure S2.

Test (using a wet universal pH indicator paper strip) above a mortar where dry AniHCl and APS has been grinded by 15 min. (left) Comparison of the strip with the color change of the indicators, suggesting a pH equal or below 1, due to HCl release. (right)

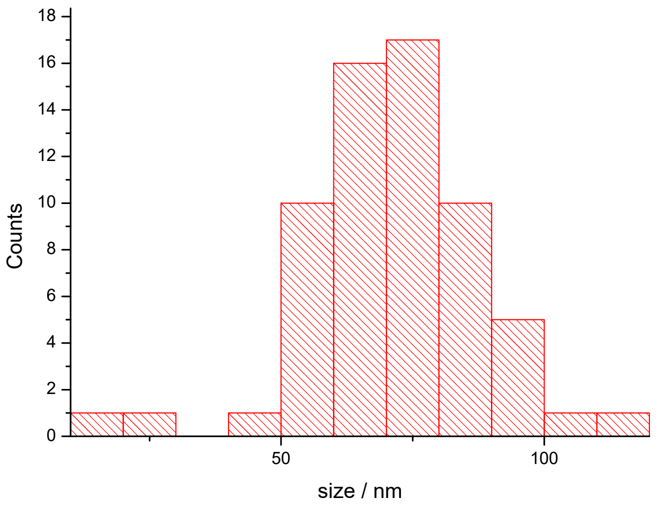

Figure S3.
Histogram of nanoparticle sizes measured from SEM image (Fig.3) in MCP PANI. Obtained using Image J and Origin®.

Experimental details

- Mechanochemical polymerzation of aniline in a mortar.

  Anilinium hydrochloride was synthesized by reacting HCl vapor (from a 37% HCl solution) with aniline (puriss. Sigma-Aldrich) in a closed plastic chamber (48 hs). The solid is subjected to dynamic vacuum al 25 oC for 48 hs. Then is reduced to fine powder in a helix mill and dried for 72 hs over anhydrous $CaCl_2$.

  Ammonioum persullfate (Aldrich) is reduced to fine powder in a helix mill and dried for 72 hs over anhydrous $CaCl_2$.

  Dry powders (0.01 moles each) are mixed gently in a plastic tube using a vortex. Then, it is placed in a glass mortar and the temperature is taken with an infrared thermometer (Etekcity). The solid is grinded with a glass pestle for determined times, photographs and temperature measurement are taken at the end of the grinding periods. Protective equipment (nitrile gloves, face mask and respirator) is used during open grinding.

  Dry resting: the lower part of a plastic chamber is filled (2 cm) with anhydrous $CaCl_2$ and left 2 hs closed for equilibration before placing the mortar inside the chamber.

  Wet resting: the lower part of a plastic chamber is covered with abosbing paper dripping wet. The chamber is left closed for 2 hs for equilibration, before placing the mortar.

The solid is rested in the mortar for 24 hs. Then, the solid is mixed with aboslute ethanol and the dispersion filtered with suction, washed with 1 M HCl and then ethanol (95%). The PANI-MCP (ES) is dried at ambient temperature over silica gel for 72 hs.

- Solution polymerization of aniline

  Aniline (puriss. Sigma-Aldrich) (0.01 moles) is dissolved in 1 M HCl and placed in a PP container with a magnetic stirring bar. To the solution are 0.01 moles of added mmonium persulfate (Aldrich), dissolved in 10 ml of distilled water. The reactor is immersed (to a level higher than the solution) in an ice bath. The temperature is monitored with a thermocouple. The clear solution becomes blue and after 30-40 min, the temperature show a small peak. After the temperature decreased to the initial value, the solution is filtered with suction and washed with 1 M HCl and ethanol (95 %). The PANI-SP (ES) is dried at ambient temperature over silica gel for 72 hs.

- SEM micrographs

  The SEM pictures were taken of dry PANI (ES, prepared by both methodds) that was grinded to fine powder and set on carbon tape. The SEM was taken with a Carl Zeiss EVO at high vacuum, without metallization. The image processing was performed with ImageJ.
